# Predicting the unpredictable: analysing the entropy and spatial distribution of ball movement patterns in field hockey

**DOI:** 10.5114/biolsport.2023.118018

**Published:** 2022-07-21

**Authors:** Felicity Lord, David B Pyne, Marijke Welvaert, Jocelyn K Mara

**Affiliations:** 1 University of Canberra Research Institute for Sport and Exercise (UCRISE), University of Canberra, Canberra, Australia; 2 Statistical Consulting Unit, The Australian National University, Canberra, Australia

**Keywords:** Performance analysis, Strategy, Sports analytics

## Abstract

Analysing the ball movement patterns of team invasion sports provides practical insight into successful strategies by identifying how and where to move the ball to create goal scoring opportunities. The aim of this study was to analyse the entropy and spatial distribution of ball movement patterns in international field hockey teams. A notational analysis system was developed in SportsCode to analyse 131 matches (n = 57 men, n = 74 women) from the 2019 Pro League tournament. The start and end location of each ball movement and the outcome of each play was recorded. Calculated variables included game possession (%), entropy, possession per zone (%) and progression rates. Decision trees identified that higher circle possession and direct movements to goal from deep attack, and lower build attack and build defence entropy, were the strategies most likely to lead to goal shots. However, teams should be unpredictable when the opposition are organised to maintain possession and unbalance the defence. Match context only had small effects on ball movement strategies highlighting there is more than one way to be successful. Executing strategies that exploit these factors should lead to greater attacking opportunities and success. Analysing the dynamic, complexity of international hockey allows coaches to prepare specific strategies for individual teams.

## INTRODUCTION

Identifying the ball movement patterns of team invasion sports is a key part of performance analysis. Performance analysis is used to provide objective insight into tactics and strategy to understand how teams are successful. Strategies are plans developed pre-game by a coach on how a team intends to attack and defend, while tactics are plans developed in-game in response to the opposition’s strategy and the evolving game situation [1]. In international women’s field hockey, transferring the ball through the wider channels (or edges) of the field and through the side of the circle, before moving the ball to the top of the circle, was identified as the most likely play leading to a goal scoring opportunity [2–3]. The most likely way to score a goal from a penalty corner was via a drag flick from the top of the circle for males, and using a pass to generate a deflection close to goal for females [4]. This type of information is critical for coaches to understand so they may implement and execute effective strategies that increase the chance of team success.

In team invasion sports, there is a delicate balance between being organised and consistent, and being disorganised and creative in ball movement patterns [5]. Unpredictability is linked to success as it creates uncertainty in the defence, whereas consistently utilising the same tactics allows the opposition to prepare counter strategies to impede this movement [5]. For example, higher values of entropy (a measure of unpredictability) were observed in basketball matches with a large deficit win compared to small deficit wins and large deficit losses [6]. This outcome implies that the degree of unpredictability can positively influence a match outcome [6]. Identifying ball movement patterns and the consistency or unpredictability of these patterns, provides insight into a team’s game style and their adaptability to match context. Game styles are defined as the consistent attacking and defensive strategies implemented by a team [7]. Game styles have previously been developed in soccer, Australian Football, Rugby Union, and field hockey by studying ingame events and describing the game actions used, strength in attack types or speed of play [8–12]. For example, Lord et al., [12] identified game styles in international field hockey teams based on whether a team typically used passing or dribbling actions, were strong or poor in established and counter attacks, created high or low set pieces per game and employed a possession or direct tempo. This analysis method identified ways a team was successful by focusing on what actions and events were occurring in a game in relation to the opposition. A similar approach can be taken to develop game styles from ball movement patterns, but instead placing greater focus on the spatio-temporal variables to understand how and where a team moves the ball to be successful. Associating the tempo and control of a team with the areas they typically play in provides critical information for developing strategies, styles and tactics. A coach can use these insights to determine which areas of the field their team needs to control, and the type of defensive structure to implement based on the consistent or unpredictable nature of the opposition.

Research investigating movement patterns in team invasion sports has increased in recent years given improvements in technology. Player and ball tracking can occur in real time using semiautomated vision-based tracking systems, or a local (LPS) and global positioning system (GPS). However, tracking technology can be expensive, and access to one team’s GPS data only provides “half the story”. Therefore, these techniques are largely restricted to professional teams. Alternatively, Stockl and Morgan [3] tracked the ball movements of hockey teams manually to identify the most likely pattern leading to a goal scoring opportunity. However, they only focused on the spatial distribution of possession, and consequently two teams may have similar profiles, but achieve different outcomes. Analysing spatial distribution alone does not provide insight into how a team moved across the field, how quickly they progressed the ball, nor how predictable they were in their movements. The combination of these variables provides a holistic perspective of strategy, and can differentiate the attacking abilities of teams.

There is an opportunity to develop new methods of analysing ball movement patterns with the resources available in field hockey that provides practical insight into performance. The primary aim of this study was to analyse the entropy and spatial distribution of ball movement patterns of international hockey teams. The secondary aims were to assess the influence of match context on these movement patterns, and identify the most common attacking movement patterns related to play outcomes.

## MATERIALS AND METHODS

Video footage of matches from the 2019 International Hockey Federation (Fédération Internationale de Hockey, FIH) Pro League (n = 57 men, n = 74 women) were accessed and analysed for this study. The tournament was an invitational competition played in a home and away season by 8 (men) and 9 (women) teams ranked in the top 12 FIH teams. Teams included Argentina, Australia, Belgium, China (women only), Great Britain, Germany, Netherlands, New Zealand, Spain (men only) and United States of America (women only). Ethical approval was granted by the Universities Human Research Ethics Committee.

**FIG. 1:**
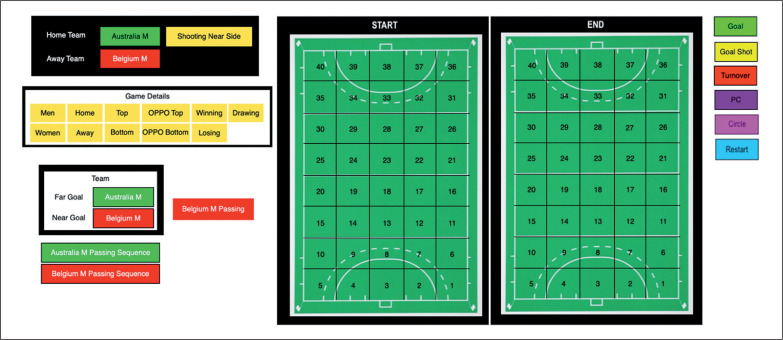
Notational analysis system used to capture ball movement patterns in SportsCode showing an example game between Australia and Belgium men’s teams. An analyst would choose the team in possession, the start and end location of each ball movement followed by the outcome at the end of a play. The field orientation would switch direction depending on which team was in possession so cell 1 always corresponded to the attacking 25 left corner and cell 40 to the defensive 25 right corner of the field

Computerised performance analysis software, SportsCode (Version 11, Hudl, https://www.hudl.com), was used to retrospectively analyse the match video footage. Variables related to the team in possession, ball movement in general play (i.e. not set pieces), the outcome of the play, and the match context, were annotated using a specifically designed code window (Figure 1). To track ball movement, the field was divided into 5 × 8 cells (i.e. 40 field zones in total). Each cell on the field that a player had control of the ball was annotated. If a player dribbled the ball through multiple cells each individual cell the ball was controlled through was notated. However, when passing, only the cells where the ball was passed from, and received, were notated.

The outcome was also recorded at the end of the possession and included a goal shot, penalty corner, restart (long corner or ball played from within the circle to outside the circle and possession retained), or turnover. Only the initial outcome was recorded; for example, if a goal shot resulted in a turnover only a goal shot was recorded, to ensure the pattern of play was analysed rather than execution of the goal shot. If a goal shot from general play or a penalty corner resulted in a rebound for the attacking team, a ball movement would not be recorded to limit movement patterns to the creation of goal scoring opportunities. A new movement pattern was only initiated if a team retained possession after a goal shot or penalty corner once the ball had left the attacking circle.

Contextual variables were recorded for each game including match status at the time of possession (winning, losing or drawing) and team ranking at the end of the tournament (1^st^ – 8^th^/9^th^). Relative team quality was calculated retrospectively by subtracting the reference team rank from the opposition team rank. For example, a relative team quality of +7 or -3 indicated a team was ranked 7 places higher and 3 places lower than the opposition.

A pilot study was completed on a randomly selected game from the database to ensure all necessary variables were recorded and the size of the cells in the code window were appropriate. Intra- and inter-coder reliability tests were also conducted on one random game to ensure coding was completed consistently and accurately. The intra- and inter-coder Kappa values were calculated as 0.92 and 0.91 for start locations, 0.91 and 0.87 for end locations and 1.0 and 1.0 for outcomes, respectively. These values indicate “very good” levels of agreement between observers [13] and the notational analysis process was deemed reliable.

### Data Analysis

**FIG. 2:**
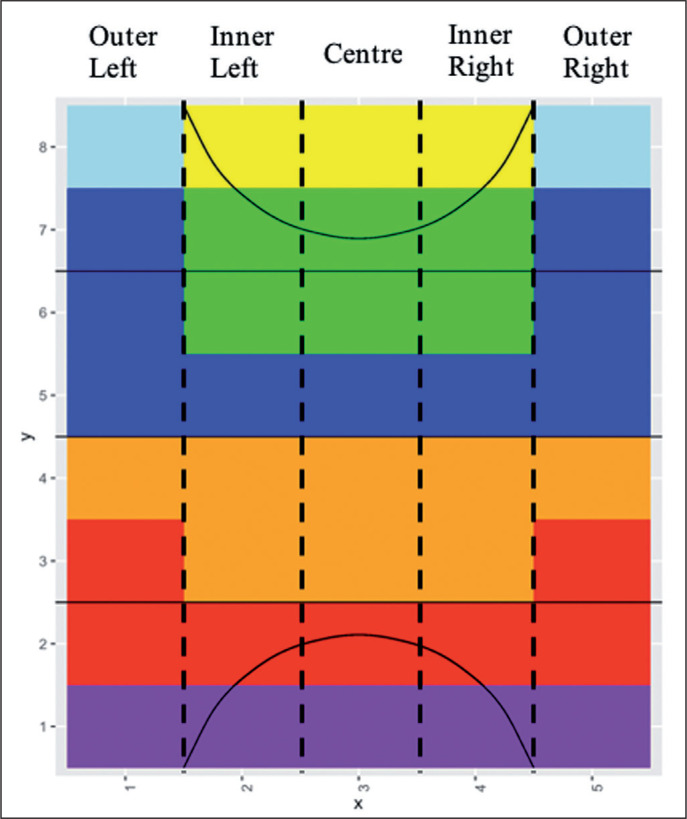
Field split into seven attacking zones; yellow = circle, light blue = corners, green = deep attack, dark blue = build attack, orange = build defence, red = outlet, purple = deep defence

The XML file for each game, which contained all the variables recorded in chronological order, was exported from SportsCode^TM^ (Version 11, Hudl, https://www.hudl.com) and converted to CSV using Microsoft Excel^TM^. These CSV files were then imported into RStudio 1.3.1093 (RStudio Inc, https://www.rstudio.com) for data analysis using R (version 4.03) [14]. Each team per match per match status (winning, losing or drawing) was analysed individually to identify how and where they moved the ball under different match situations. The 40 cells were grouped into 7 attacking zones based on the different phases of attack occurring in each zone. Table 1 describes the definitions for each attacking zone and their respective phases of attack, and Figure 2 illustrates these zones on the field.

**TABLE 1:** Attacking zone definitions relating areas on the field to phase of attack

Attacking Zone	Location	Aim	Players and ball movement
Circle	Areas closest goal encompassing the majority of the circle	Create goal scoring opportunities	Attackers attempt to move the ball along the baseline to the centre of circle as this is the best goal scoring position given the angle and proximity to goal.
Corners	Two corners of the attacking 25 bordered by the baseline and sideline	Create circle entry	Safer path into the circle as the offence look to move the ball around the edge of the defence along the baseline into the circle. However, the longer route into the circle provides greater opportunities for the defence to get numbers to better goal scoring positions.
Deep Attack	Centre of the attacking half and top of the circle	Create circle entry and/or goal scoring opportunity	Attackers attempt to move directly to the top of the circle for a goal shot or pass closer to goal, providing the more direct route to goal and best angle for a goal shot. However, it is also a riskier option as a team must play through the defenders clustered around the circle, increasing the chances of a turnover and counter attack.
Build Attack	Edges of the attacking half and across the attacking side of the half way line	Manipulate defence to create space for an attacking opportunity	Attack look to manipulate the defence by passing back and around the midfield. Defence condense into their defensive half, protecting the direct routes to goal. The depth and width of this shape reflects the spread of the attackers in an attempt to maintain possession by transferring the ball backwards or across the field.
Build Defence	Defensive side of the half way line and centre of the defensive 50	Gain ground, manipulate defence	Until ground can be gained defenders transfer the ball from side to side to attempt to generate holes in the opposition’s structure. Generally, a team will have one or two central defenders and two wide defenders reflecting the shape of this zone.
Outlet	Areas used to take free hits from the defensive 25 and the likely path out from defence	Gain ground	Defence are set up in front of the ball, protecting the centre but allowing the player to move to the sideline. Attackers generally have two central defenders and two higher wider defenders for moving the ball up the sideline.
Deep Defence	Area along the baseline of the defensive 25	Protect goal	Danger zone for attacking team, turnover in this area could lead to a goal shot or restart of play for the opposition. Attackers look to move wide towards the space near the sideline to remove ball from circle and away from the defence.

Data were analysed for entropy and spatial distribution which included game possession, possession per zone, and progression rates (i.e. the percentage of ball movements in different directions). Time in possession was calculated by summing the total time of all actions completed per cell location by calculating the difference between start and end times for each movement. The percentage of game possession for a team in a single match was calculated by dividing the total time for the reference team’s game actions by the total time for both team’s game actions per match. This metric reflected how much control a team had per match. Possession per zone was calculated by dividing the time for all game actions in one attacking zone by the total time in possession for all attacking zones for a team. This metric indicated the offensive opportunities of a team.

Shannon’s entropy (*H*) was calculated to determine the unpredictability of ball movement for each cell location [6], which reflects the passing range of a team and the variety of ways they may attack. The equation for Shannon’s entropy is shown in Equation 1 where *p*_i_ is the probability of the ball entering a certain location, and *n* is the total number of locations the ball can enter [15]. The minimum entropy value of 0 indicates a highly predictable movement pattern. The maximum entropy value is determined by taking the natural logarithm of the number of locations included in the calculation. If the ball is transferred from one cell to every other location on the field, then *n* is equal to 40. Therefore, the theoretical maximum entropy for this analysis was 3.69 (log_e_(40)). The mean was then calculated for each attacking zone. Entropy values were normalised by dividing by the theoretical maximum (3.69) entropy, so the theoretical minimum normalised entropy value was 0 and the theoretical maximum normalised entropy was 1.

Equation 1: $H(X)=H(p_{1},\ldots ,p_{n})=-\sum_{i=1}^{n}p_{i}log_{e}p_{i}$

Progression rates were calculated to determine the intent of teams to attack directly and progress the ball towards goal, or maintain possession. A novel method was developed to assess progression rates. Progression rates were calculated for each of the 40 cells by calculating the percentage of ball movements that ended in each cell. Cells were then grouped into the 7 attacking zones, for start and end locations, and averages calculated for each zone. Movements were simplified to four direction categories so they could be compared between zones. Direction categories included:

– Back: ball movement was received in a zone behind the starting zone
– Stay: ball movement started and ended within the same zone
– Forward: ball movement was received in the adjacent zone in front of the starting zone
– Goal: ball movement ended in a zone 2 or 3 ahead from a starting zone in the defensive half, or ended in a zone more direct to goal through the centre of the field from the attacking half

Table 2 shows the description of categories for each attacking zone. Attacking team profiles were developed by analysing when a team was in attack, and defensive team profiles by calculating opposition values for each team.

**TABLE 2:** Movement progression categories for each attacking zone representing the least to most direct attacking options to take from left to right. For example, a team that has possession in the Circle (first row below), if they move into the Corners or Build Attack zones it is considered a movement back, if they dribble or pass within the circle the team has not changed zones so it is considered as a stay movement, and if the team moves the ball to the Deep Attack zone it is considered to be movement in the forward direction as the ball remains in an attacking position for a goal shot, even though the initial ball movement is away from goal.

Directness Start Zone	Least Back	Stay	Forward	Most Goal
Circle	Corners Build Attack	Circle	Deep Attack	NA

Corners	Build Attack Build Defence	Corners	Deep Attack	Circle

Deep Attack	Build Attack Build Defence	Deep Attack	Corners	Circle

Build Attack	Build Defence Outlet	Build Attack	Corners	Deep Attack Circle

Build Defence	Outlet Deep Defence	Build Defence	Build Attack Corners	Deep Attack Circle

Outlet	Deep Defence	Outlet	Build Defence	Build Attack Deep Attack

Deep Defence	NA	Deep Defence	Outlet	Build Defence Build Attack

### Statistical Analysis

Statistical analysis was completed in RStudio separately on male and female data. Data were transformed to a z-score to ensure equal weighting in the analysis. Linear mixed models, using the ‘*lmer*’ function from the *‘lme4’* R package [16], were used to analyse the effect of contextual variables and attacking zones on game possession, entropy, possession per zone and progression rates. In the first model, match status and attacking zones were identified as fixed factors, and team and game number included as nested random factors, and relative team quality as a random factor. In the second model, relative team quality and attacking zones were identified as fixed factors and team, game number and match status included as nested random variables. The interaction between fixed factors was analysed in both models. Assumptions of normality and homoscedasticity were checked via visual inspection of residual plots and histograms.

Likelihood ratio tests, using the *‘anova’* function, were used to determine the statistical significance of a fixed or random factor by comparing models with and without the variable in question. To identify significant pairwise comparisons in a model assessing match status, the ‘*emmeans*’ function with false discovery rate correction [17] from the ‘*emmeans’* R package [18] was employed. To assess relative team quality, the *‘emtrends’* function from the ‘*emmeans’* R package [18] was used. Results are presented as unstandardised estimated marginal mean ± 95% confidence limits (CL) and considered significant when p < 0.05. Practical differences were identified by considering the aim of the attacking zone, and the minimal difference in possession, entropy or progression rates needed to achieve this aim.

Decision trees were developed, using the ‘*rpart*’ function from the ‘*rpart’* R package [19], to assess the variables determining the outcome of a play – goal shot, positive (penalty corner or restart), or turnover. Forty predictor variables were included in the model including entropy, possession, and movements back, stay, forward and goal for each attacking zone. The prediction accuracy of the model was assessed by splitting the data into train (70%) and test (30%) data sets and using the *‘predict’* function from the *‘caret’* R package [20]. The overall accuracy was 86% for men and 91% for women with sensitivity (true positives) > 79% and specificity (true negatives) > 88%. The decision tree model was deemed able to accurately distinguish play outcomes. The *‘varImp’* function from the *‘caret’* R package [20] was used to identify which variables distinguished play outcomes.

## RESULTS

### Match Status

The total number of ball movements recorded for men in 57 games when winning was 18406, losing 18799, and drawing 22680. The mean number of ball movements completed per team per game when winning was 301 (± 398) (mean ± standard deviation), losing 261(± 143) and drawing 201 (± 122). The mean length of time in seconds of each ball movement when winning was 2.16 s (± 1.30 s), losing 2.14 s (± 1.24 s) and drawing 2.14 s (± 1.23 s). For women, the total number of ball movements recorded in 74 games when winning was 20147, losing 19818 and drawing 26621. The mean number of ball movements completed per team per game when winning was 221 (± 143), losing 218 (± 129) and drawing 180 (± 112). The mean length of time in seconds of each ball movement when winning was 2.12 s (± 1.24 s), losing 2.09 s (± 1.15 s) and drawing 2.08 s (± 1.16 s).

**FIG. 3:**
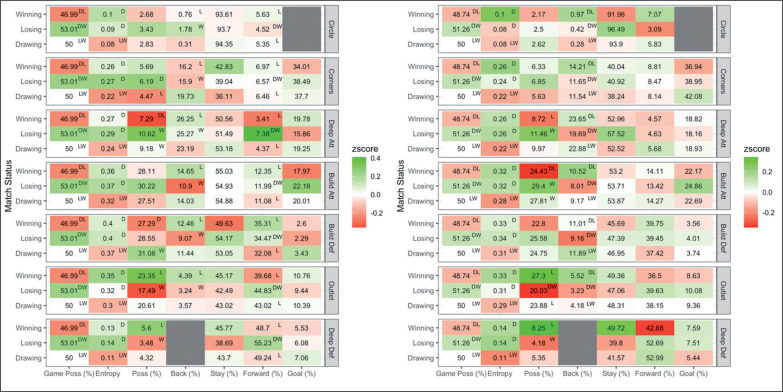
A comparison of ball movement patterns when winning, losing and drawing for men’s (left) and women’s (right) teams in the 2019 Pro League tournament. Results are presented as average of ball movement variable on the x axis per match status per attacking zone on the y axis. Z-score indicates above (green) and below (red) average performance compared to the league average. D = significant difference compared to drawing, L = significant difference compared to losing, and W = significant difference compared to winning teams. For example, game possession for men, losing teams have greater possession than drawing and winning teams and drawing teams have greater game possession than winning teams.

Men’s and women’s profiles when winning, losing and drawing and pairwise comparisons are presented in Figure 3. For both men and women, practical differences were observed between teams for game possession and possession per zone. For men, losing teams had greater game possession (8.1 ± 1.7%; mean ± 95% CL) and greater possession (3.3 ± 3.0%) in deep attack than winning teams. Winning teams had greater possession (5.9 ± 5.2%) in outlet than losing teams. For women, losing teams had greater game possession (6.1 ± 1.1%) and greater possession in build attack (5.0 ± 4.5%) than winning teams. Winning teams had greater possession in deep defence (4.1 ± 3.9%) and outlet (7.3 ± 6.0%) than losing teams.

### Team Quality

For men, the total number of ball movements recorded when relative team quality was -7 was 893 (2 games), +1 was 8683 (16 games) and +7 was 1255 (2 games). The mean number of ball movements per team per game when relative quality was -7 was 447 (± 8), +1 was 548 (± 88) and +7 was 628 (± 43). The mean length of time in seconds of each ball movement when relative quality was -7 was 2.05 s (± 1.18 s), +1 was 2.06 s (± 1.17 s) and +7 was 2.08 s (± 1.17 s). For women, the total number of ball movements recorded when relative team quality was -8 was 774 (2 games), +1 was 7702 (17 games) and +8 was 1291 (2 games). The mean number of ball movements per team per game when relative quality was -8 was 387 (± 23), +1 was 453 (± 50) and +8 was 646 (± 15). The mean length of time in seconds of each ball movement when relative quality was -8 was 1.98 s (± 1.06 s), +1 was 2.08 s (± 1.19 s) and +8 was 1.89 s (± 1.05 s).

**FIG. 4:**
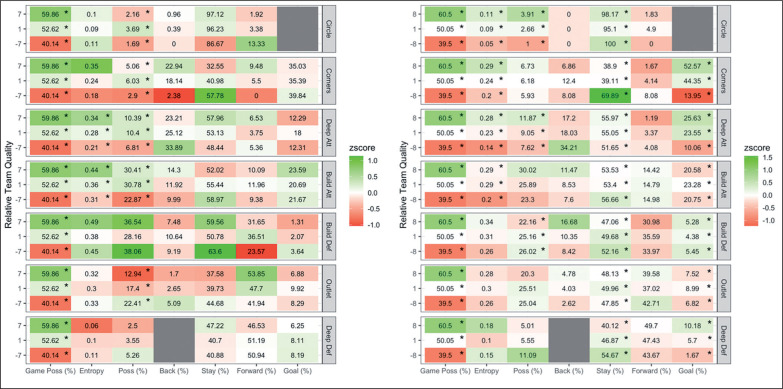
A comparison of ball movement patterns when relative team quality is +7, +1 and -7 for men’s (left) and +8, +1 and -8 for women’s (right) teams in the 2019 Pro League tournament. Results are presented as average of ball movement variable on the x axis per match status per attacking zone on the y axis. Z-score indicates above (green) and below (red) average performance compared to the league average. * indicates significant trend in relative quality of teams. For example, game possession for men decreases with ranking from teams ranked 7 places higher than their opposition to teams ranked 7 places lower

Men’s and women’s profiles are shown for lower, similar and higher ranked teams (relative to the reference team) in Figure 4. For men, game possession increased by 1.4% (± 0.6%) with an increase in team quality by one ranking (place). Entropy increased by 0.0057 (± 0.0053) in deep attack and by 0.0062 (± 0.005) in build attack with an increase in ranking. Possession increased by 0.4% (± 0.3%) in build attack, and decreased by 0.8% (± 0.3%) in outlet with an increase in ranking by one.

For women, practical differences were observed in game possession, an increase of 0.9% (± 0.4%) was seen with an increase in one ranking (place). As ranking increased, entropy in deep attack increased by 0.0065 (± 0.0041). Ball movements that stayed in the corners were higher for lower ranked teams. Movements directed towards goal were higher for relatively higher ranked teams from corners and deep attack.

### Play Outcomes

The total number of ball movements recorded in 57 games for men during phases of play leading to a goal shot was 6401, a positive outcome 8808 and a turnover 44255. The mean number of ball movements completed per team per game resulting in a goal shot was 56 (± 30), a positive outcome 78 (± 36) and a turnover 388 (± 58). The mean length of time in seconds of each ball movement in plays resulting in a goal shot was 1.98 s (± 1.18 s), a positive outcome 2.08 s (± 1.22 s) and a turnover 2.18 s (± 1.27 s). The total number of ball movements for women in 74 games resulting in a goal shot was 5657, a positive outcome 7993 and a turnover 52309. The mean number of ball movements per team per game for plays resulting in a goal shot was 38 (± 24), a positive outcome 54 (± 24) and a turnover 353 (± 44). The mean length of time in seconds of each ball movement for plays resulting in a goal shot was 1.87 s (± 1.06 s), a positive outcome 2.08 s (± 1.16 s) and turnover 2.12 s (± 1.19 s).

**FIG. 5:**
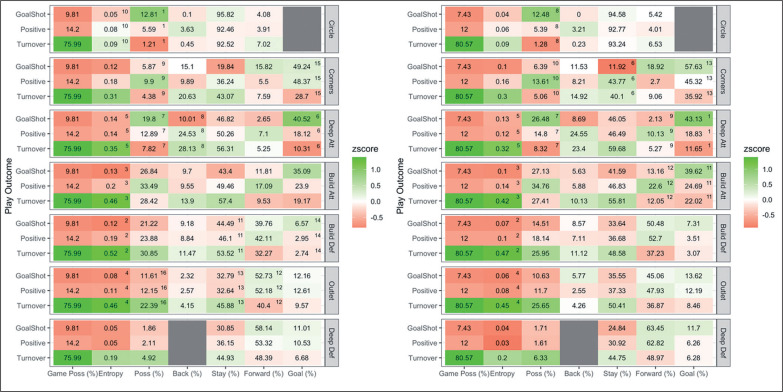
A comparison of plays ending in a goal shot, positive or turnover for men’s (left) and women’s (right) teams in the 2019 Pro League tournament. Results are presented as average of ball movement variable on the x axis per play outcome per attacking zone on the y axis. Z-score indicates above (green) and below (red) average performance compared to the league average. Superscripted numbers indicate variables important in distinguishing play outcomes in order of importance. For example, circle possession was the most important variable for men, with plays ending in a goal shot having greater circle possession than positive outcomes and positive outcomes having greater circle possession than turnovers.

Men’s and women’s profiles when a play ends in a goal shot, positive outcome, or a turnover, and the variables important in distinguishing play outcomes are shown in Figure 5. A total of 16 variables were considered important for men, with the three highest-ranked variables including Circle Possession, Build Defence Entropy and Build Attack Entropy. For women, a total of 13 variables were considered important, and the three most important variables were Deep Attack Goal, Build Defence Entropy and Build Attack Entropy.

### Team Profiles

**FIG. 6:**
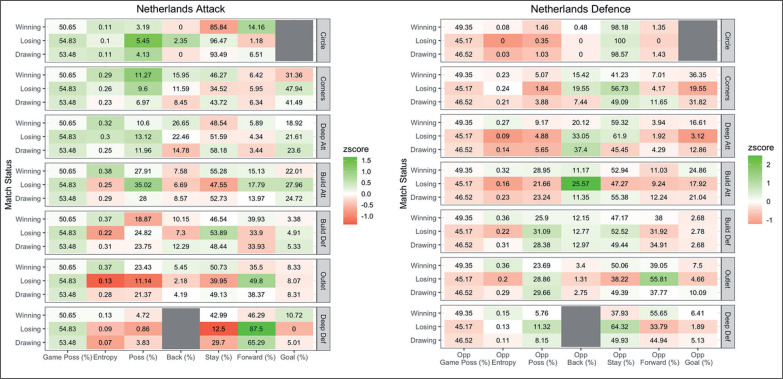
Attacking (left) and defensive (right) ball movement variables for Netherlands women’s team when winning, losing or drawing. Results are presented as average of ball movement variable on the x axis per match status per attacking zone on the y axis. Z-score indicates variables used more (green) and less (red) often than the league average.

An example profile for Netherlands women is displayed in Figure 6 for attacking and defensive variables when winning, losing and drawing. Additional supplementary files showing all individual team profiles are available from the corresponding author.

## DISCUSSION

This study analysed the entropy and spatial distribution of ball movement patterns in international field hockey. Ball movement profiles were developed by analysing the interaction of these spatio-temporal variables. This novel method of data capture and analysis provides more practical insights into performance as it accounts for the multitude of factors influencing ball movement patterns. To be successful, teams need to be both predictable when direct opportunities to attack arise, but also unpredictable when trying to maintain possession in attacking positions and unbalance the defence. The results of this study highlight the importance of analysing the critical factors influencing strategy. This approach ensures that coaches have the level of detail needed to develop specific strategies for success in different match situations.

Differences in ball movement variables were evident between play outcomes in the FIH Pro League, and similar trends were observed between genders highlighting several basic principles of hockey. A turnover was likely to occur when a play had greater entropy, higher possession in the defensive half, and a greater proportion of actions staying in defence or moving back in attacking positions. In contrast, a positive outcome resulted from plays with lower entropy, but higher possession in corner zones as teams had a greater proportion of actions moving forward. Goal shots, also resulted from plays with lower entropy, but higher possession in deep attack and the circle, and a greater proportion of actions moving more directly to goal. The ability to progress the ball quickly and more directly through the centre of the field provides greater opportunities for goal shots before the opposition can organise their defence. An inability to penetrate the defence and progress directly to goal allows greater time for the defence to protect the direct lines to goal, subsequently more likely resulting in a turnover when a team is held up in the defensive half and a positive outcome in attacking positions. This outcome reflects earlier research indicating a goal scoring opportunity was more likely if the ball was possessed near, or at the top of the circle [3], and a goal scored the further up the pitch the ball was regained [2]. Understanding differences between these play outcomes, assists both the analyst and coach interpret team profiles and assess the attacking ability of a team.

Hockey is a low scoring game and our results show > 75% of game possession were related to plays ending in a turnover. Thus, goal scoring opportunities are likely to result from a sequence of plays that aim to disorganise the defence by building pressure in attacking positions, while restricting the opposition doing the same. For both men and women, teams ranked relatively higher had greater game possession, and entropy and possession in zones in the attacking half. Although these effects were small, they reflect the trend for greater differences in attacking opportunities as the relative team difference increased given the ability of higher ranked teams to control the ball in the attacking half. A limitation of this study was that all teams were ranked within the top 12 international teams in 2019 which may have masked the impact of relative team quality on ball movement patterns. A larger effect may have been seen if there were teams competing from a greater range in rankings. Nonetheless, this outcome is consistent with analysis in soccer where the top-ranked teams completed greater actions related to ball possession and ending actions [21], had the greatest mean values of entropy [22], and greater width and length in attack against weaker opposition [23]. This pattern of play makes higher ranked teams more unpredictable and harder to defend against as the opposition has to protect a larger portion of the field. Consequently, higher ranked teams are more likely to create greater attacking opportunities per game.

When analysing the effect of match status on ball movement variables for both men and women, compared to teams that are drawing, losing teams have more game possession, greater entropy and possession in the attacking half. While winning teams have less game possession, but greater entropy and possession in the defensive half as they are more likely to move backwards. Although these effects may be small, they reflect a tendency for teams to change strategy when there is a change in match status. A larger effect may be observed if score differences and interaction with relative team quality are assessed. Nonetheless, winning teams are more likely to retreat to their defensive half to protect their goal, while losing teams are likely to increase their defensive pressure higher up the ground. This pattern is observed in soccer, with losing teams having greater possession in the attacking half, and more likely to increase their use of build-up and sustained threats plays which increased a team’s level of game possession, and winning teams having greater possession in the defensive half, and increased use of direct attacks [24, 25]. The relationship between entropy and possession reflects the change in strategy with match status that increases the attacking opportunities of losing teams, while reducing those of winning teams.

It appears there are two key factors related to success which a coach can use to develop effective strategies. First, it is the ability of a team to get the ball to the build attack zone quickly, and secondly maintaining possession in the attacking half using a variety of movement patterns, that increases the chance of the play being successful. We have showed that successful hockey teams and plays revolve around possession in the attacking half, utilising the length and width of the attacking half to disorganise the defence and create space to attack the direct routes to goal. This is reflected in research identifying soccer teams were more likely to create a goal scoring opportunity from a counter attack or regaining the ball in the attacking third [26]. In basketball only front (attacking) court entropy, as opposed to full court entropy was related to success [6], and entropy was positively correlated to generating future high probability shots [27]. Therefore, teams may be predictable when the opposition is disorganised, and immediate scoring opportunities present themselves, but unpredictable when the opposition are organised to maintain possession while attempting to unbalance the defence.

The relatively small effect of match status and team quality on ball movement patterns may reflect the different strategies individual teams use in different situations to be successful. For example, Netherlands (women) are a dominant team as they have high game possession, and entropy and possession in the attacking half while limiting their oppositions attacking possession by forcing them backwards. In contrast, Australia (women) have lower game possession, lower entropy in the attacking half and lower circle possession, and concede high opposition possession in their attacking half. However, Australia’s ability to absorb pressure and counter attack led them to the final of the 2019 Pro League tournament against Netherlands. The opposing strategies reflects the need to analyse teams individually as there is more than one way to win a game of hockey and teams must develop strategies that reflect the abilities of their players.

## CONCLUSIONS

We developed attacking and defensive ball movement profiles in international field hockey using video and notational analysis of the 2019 FIH Pro League men’s and women’s tournaments. These profiles were developed by analysing the entropy, possession and progression rates of ball movement patterns and assessing the interaction of these spatio-temporal variables across seven attacking zones. These profiles highlight the direction and unpredictability of a team’s ball movements and the attacking opportunities that arise. The ability to quickly get the ball to and then maintain possession in the attacking half using a variety of patterns are key factors in developing effective strategies. This novel approach provides important evidence for coaches to develop strategies that utilises their team’s strengths and exploits the opposition’s weaknesses.
